# Using light to tell the time of day: sensory coding in the mammalian circadian visual network

**DOI:** 10.1242/jeb.132167

**Published:** 2016-06-15

**Authors:** Timothy M. Brown

**Affiliations:** Faculty of Life Sciences, University of Manchester, Manchester M13 9PT, UK

**Keywords:** Melanopsin, Suprachiasmatic, Vision

## Abstract

Circadian clocks are a near-ubiquitous feature of biology, allowing organisms to optimise their physiology to make the most efficient use of resources and adjust behaviour to maximise survival over the solar day. To fulfil this role, circadian clocks require information about time in the external world. This is most reliably obtained by measuring the pronounced changes in illumination associated with the earth's rotation. In mammals, these changes are exclusively detected in the retina and are relayed by direct and indirect neural pathways to the master circadian clock in the hypothalamic suprachiasmatic nuclei. Recent work reveals a surprising level of complexity in this sensory control of the circadian system, including the participation of multiple photoreceptive pathways conveying distinct aspects of visual and/or time-of-day information. In this Review, I summarise these important recent advances, present hypotheses as to the functions and neural origins of these sensory signals, highlight key challenges for future research and discuss the implications of our current knowledge for animals and humans in the modern world.

## Abstract (precis)

**Summary:** Accurately predicting daily changes in the environment is critical for health and survival. Mammals use surprisingly sophisticated sensory processing mechanisms to achieve this task.

## Introduction

The ability to predict recurring changes in the environment is critical to survival, allowing organisms to maximise opportunities to secure food, reproduce and avoid danger. For almost all species, the most relevant environmental changes are those associated with the earth's daily rotation. Accordingly, virtually all organisms possess internal circadian clocks which allow them to anticipate day–night transitions and adapt their physiology and behaviour accordingly. The significance of such processes is well recognised, with evidence from bacteria to insects and mammals indicating that impaired circadian function is associated with adverse effects on health and survival (DeCoursey et al., 2000; Ouyang et al., 1998; Pittendrigh and Minis, 1972; Saunders, 1972; Spoelstra et al., 2016). In particular, such studies highlight the importance of an appropriate relationship between internal timing and the external environment. As such, because light provides the most reliable source of information about time of day, photoreceptive input is an essential regulator of the circadian system in almost all organisms.

The influence of light on circadian timing (photoentrainment) has been studied in many species (Pittendrigh, 1988). Although there is remarkable homology in the mechanisms employed across organisms, this Review will primarily focus on mammals. Unlike all other species studied thus far, in mammals there is a clear separation between the site of photoreception and the principle circadian clock. Hence, mammalian circadian photoreception occurs only in the retina (Czeisler et al., 1995; Meijer et al., 1999; Nelson and Zucker, 1981), from where visual signals are conveyed by direct and indirect neural projections to the hypothalamic suprachiasmatic nucleus (SCN), site of the master circadian pacemaker (Morin and Allen, 2006). Neural outputs from the SCN to other hypothalamic sites responsible for autonomic and neuroendocrine regulation (Kalsbeek et al., 2006) then allow the clock to coordinate daily variations in physiology and behaviour.

Given the fundamental role of the clock in animal health, understanding the sensory signals that regulate circadian timing and the potential impact of artificial lighting on this process is now of great importance. Here, I highlight recent advances in our understanding of sensory inputs to the clock, present hypotheses as to their functional roles and, throughout the Review, highlight areas of uncertainty that constitute important challenges for future research.

Glossary **Amacrine cells**

Primarily inhibitory retinal interneurons providing a mechanism for the lateral spread of visual information and a conduit through which rod bipolar cells can communicate with retinal ganglion cells.

**Chromatic opponency**

The subtractive processing of signals originating from photoreceptor cells with different spectral sensitivities to extract information about ‘colour’. In mammals, this process originates in the retina downstream of cone photoreceptors, resulting in ganglion cells that discriminate ‘blue’ versus ‘yellow’ light (by comparing signals from short-wavelength versus medium- and/or long-wavelength-sensitive cone opsins) or ‘red’ versus ‘green’ light (in humans and certain primate species with both medium- and long-wavelength-sensitive cone opsins).

**Civil twilight**

Portions of the day during which the sun is between 0 and 6 deg below the horizon, corresponding to the approximate limit at which solar illumination allows objects to be clearly distinguished.

**Photopic**

‘Bright’ light conditions under which cone photoreceptors provide the primary source of visual information (luminance above 3 cd m^−2^, corresponding to ambient illumination above 10^12^ photons cm^−2^ s^−1^).

**Scotopic**

Low light conditions under which rods provide the sole source of visual information (below 0.01 cd m^−2^, corresponding to ambient illumination below 10^10^ photons cm^−2^ s^−1^).

**Temporal gating**

Daily changes in the sensitivity of the circadian system to photic input, such that light detected during the evening shifts rhythms later (delays), light in the morning shifts rhythms earlier (advances) and light produces no change in timing during the middle of the day.

## Sensory properties of circadian photoentrainment

### Temporal gating and integration

**Fig. 1.**
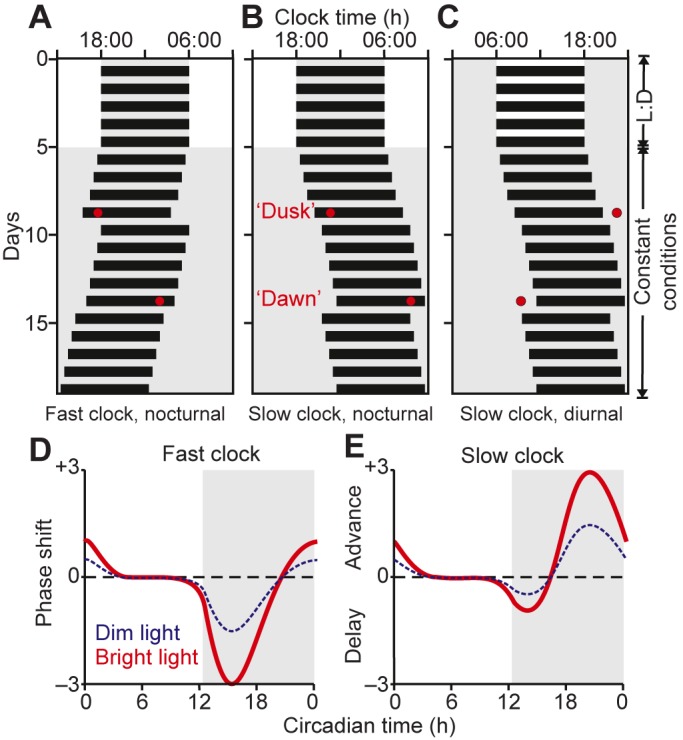
**Temporal gating and integration in the circadian system.** (A–C) Schematic ‘actograms’ showing daily activity (black bars) for nocturnal (A,B) or diurnal (C) mammals under a light:dark (L:D) cycle and following transfer to constant conditions to reveal the biological clock's intrinsic period; here either shorter (A; 23.5 h) or longer (B,C; 24.5 h) than 24 h. In each case, light pulses (red circles) presented around the animal's subjective evening (‘dusk’) delay the onset of activity on subsequent days, whereas light pulses presented during the subjective morning (‘dawn’) advance activity onset. (D,E) Phase-response curves (PRCs) quantifying the change in activity onset as a function of the circadian time at which light is detected for bright and dim light (circadian time 12 designated as the onset of subjective night). Note that PRCs for individuals with fast clocks are characterised by large delay and small advance portions (D) and vice versa for individuals with slow internal clocks (E). As such, organisms with fast clocks primarily use dusk light for entrainment (because these require a daily phase delay to bring their endogenous period up to that of the 24 h solar day), whereas organisms with slow clocks primarily use dawn light (because these require a daily advance to maintain 24 h rhythms). In all plots, periods of light are indicated by white backgrounds and periods of darkness are indicated by grey backgrounds.

Early experiments in the field investigated the effects of light on circadian patterns of voluntary activity in various nocturnal rodent species (Daan and Pittendrigh, 1976; DeCoursey, 1960, 1964). These experiments established a key principle underlying circadian photoentrainment: responses to light vary predictably depending on time of day (temporal gating; see Glossary). Hence, light exposure in the early night shifts activity to later time points (phase delays), light in the late night shifts activity to earlier time points (phase advances) and light has no effect during the middle of the day (Fig. 1). This is also true in diurnal mammals, including humans (Hoban and Sulzman, 1985; Kas and Edgar, 2000; Khalsa et al., 2003; Mahoney et al., 2001). Indeed, this mechanism is characteristic of the majority of organisms, although with certain interspecific (Pittendrigh, 1988) and intraspecific (Daan and Pittendrigh, 1976; DeCoursey, 1960) differences: the relative size of phase advances and delays depends on the endogenous circadian period – fast clocks (periods <24 h) are associated with larger delays, whereas slow clocks (periods >24 h) are associated with larger advances. This arrangement ensures a stable period and phase of the internal clock relative to the strictly 24 h solar cycle (Fig. 1).

Although temporal gating is vital for photoentrainment, it would be of little use without mechanisms for discriminating photic signals that distinguish the onset of day or night from other visual information. To ensure a stable representation of form and motion across the huge daily variations (∼9 decimal orders) in background light intensity encountered in the natural world, the visual system must adapt its sensitivity in parallel with changes in ambient light levels (Perlman and Normann, 1998). To do this, the adaptation mechanism subtracts information about ambient light intensity in order to measure relative differences in radiance across the visual scene. This kind of processing seems of little use for distinguishing time of day, however. Instead, the most useful information for the circadian clock would seem to be a direct measure of ambient light levels. Consistent with this view, the photoentrainment pathway in nocturnal rodents appears to act as a ‘photon counter’, which can integrate over extended time periods (>1 h) to produce a graded response based on the amount of light detected (Lall et al., 2010; Nelson and Takahashi, 1991, 1999; although see Vidal and Morin, 2007). It should be noted that this integration capacity is not unlimited (i.e. there is a maximal possible response dictated by the temporal gating mechanism). Nonetheless, the important features of this arrangement are that even very low levels of illumination (<10^11^ photons cm^−2^ s^−1^) can alter circadian timing given extended exposure. By contrast, the greater levels of ambient illumination encountered during twilight (∼10^12.5^–10^14.5^ photons cm^−2^ s^−1^) can powerfully adjust clock timing, even for very short exposure durations.

**Fig. 2.**
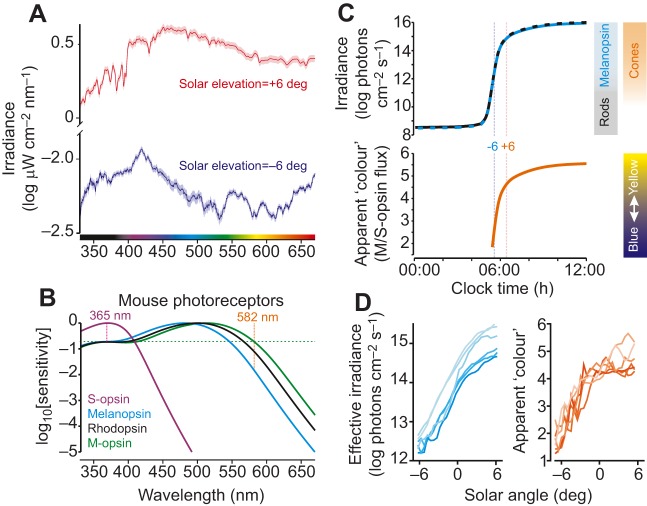
**Colour and brightness as indicators of time of day.** (A) Spectral irradiance of ‘average’ daylight measured when the sun is 6 deg above or below the horizon. Note that, in addition to the pronounced difference in the amount of light, there is also a substantial change in spectral composition, with reduced middle-wavelength (‘green-yellow’) light at negative solar angles. (B) Sensitivity profiles of the four visual opsin proteins responsible for photoreception in mice, illustrating the distinction between colour and brightness: monochromatic light at 365 and 582 nm produces identical activation of mouse M-opsin but differs in apparent ‘colour’ because the longer wavelength light provides much weaker activation of S-opsin. Importantly, 582 nm light also appears substantially ‘dimmer’ for both rods (rhodopsin) and melanopsin. (C) Top, approximate corneal irradiance, weighted according to mouse rod and melanopsin sensitivity, between midnight and midday on a typical equinox day (clear sky, no moon). Bars to the right indicate effective sensitivity ranges for rod-, melanopsin- and cone-based responses (see Lucas et al., 2012). Bottom, apparent colour of daylight for the mouse visual system (ratio of effective irradiance weighted according to M- versus S-cone opsin sensitivity), demonstrating a progressive shift towards ‘yellow’ with increasing solar elevation. Nocturnal irradiance levels (solar angles <7 deg below horizon) were estimated based on commonly reported values for clear night skies. Apparent colour under these conditions is not relevant because irradiance falls below the threshold for cone-based vision (>10 log photons cm^−2^ s^−1^). (D) Apparent rod and melanopsin irradiance (left) and colour (right) as a function of solar angle for six real dawn-to-dusk transitions. In both cases, traces are colour coded, with lighter lines corresponding to days with higher overall irradiance (i.e. less cloud cover). Note that although cloud can reduce irradiance substantially (∼10-fold here), colour remains relatively unaffected. Data in A–D are derived from the dataset presented in Walmsley et al. (2015) (collected in Manchester, UK; 31 Aug–14 Oct 2005).

Thus, the photoentrainment pathway is optimised to signal the changes in illumination associated with twilight, whereas temporal gating modulates the amplitude and direction of the responses (Nelson and Takahashi, 1991). Together, these mechanisms ensure that circadian resetting only occurs around day–night transitions. Although this conceptual model appears sufficient to explain how animals synchronise their clocks to the solar day, the amount of light is not the only source of photic information that reliably changes around twilight. Indeed, the spectral composition (colour) of light reaching the earth also exhibits predictable changes (Fig. 2A), as a result of variations in atmospheric filtering (Hulburt, 1953). The idea that animals might use this variation in colour as a circadian time cue was suggested many years ago (Roenneberg and Foster, 1997) but, owing to the technical difficulties inherent in distinguishing responses to the colour versus brightness of light, it was only recently tested in mammals (Walmsley et al., 2015; see below).

### Photoreceptive systems encoding brightness and colour

Biological photoreceptors such as the opsin:vitamin A-based photopigments used by mammals exhibit distinct genetically determined sensitivities to light of different wavelengths (Govardovskii et al., 2000). Accordingly, the response of any photoreceptive protein must depend on both the amount and spectral composition of light. Note that this arrangement is distinct from the ability to measure colour (Fig. 2B), which requires comparing the relative activity of at least two different classes of photoreceptor (Jacobs, 2014). To effectively study the importance of colour and/or ‘brightness’ for circadian photoreception, it is first necessary to determine which retinal photoreceptors provide input to the clock and their spectral sensitivities. One way to address this is to evaluate the sensitivity of the clock to varying wavelengths of monochromatic light. Unfortunately, such approaches have limited effectiveness when the candidate photoreceptors have similar spectral sensitivity, as for the nocturnal rodents (Fig. 2B) favoured for circadian studies (Provencio and Foster, 1995; Takahashi et al., 1984; Yoshimura and Ebihara, 1996). Thus, the development of transgenic mouse models lacking key photoreceptive pathways (or with altered photoreceptor sensitivity) has been a crucial step in elucidating the photoreceptive origins of circadian photoentrainment.

An especially important finding was that circadian photoreception persists in ‘blind’ (rodless/coneless) mice (Freedman et al., 1999; Lucas et al., 1999). Subsequent work then identified that the origin of this residual photoreception was a novel opsin, melanopsin, expressed by a subset of output retinal neurons (Berson et al., 2002; Gooley et al., 2001; Hattar et al., 2002, 2003; Panda et al., 2003; Provencio et al., 2000). Importantly, however, although genetic lesions of these melanopsin-expressing retinal ganglion cells (mRGCs) essentially abolish photoentrainment (Guler et al., 2008; Hatori et al., 2008), knockout of the melanopsin protein alone does not (Panda et al., 2002; Ruby et al., 2002). Thus, mRGCs act as conduits for both extrinsic (rods/cones) and intrinsic (melanopsin) photoreceptor systems to regulate circadian photoentrainment; mice lacking critical components of all three photoreceptor types are unable to entrain (Hattar et al., 2003). Indeed, we now know that all mammalian photoreceptor classes contribute to circadian photoentrainment, with each accounting for distinct aspects of the overall sensory characteristics, as discussed below.

#### Rods, melanopsin and brightness detection

Because rods are the most sensitive mammalian photoreceptors, their role in circadian photoentrainment is conceptually straightforward. Mice lacking functional rods do not reliably entrain to light levels below those typically encountered at civil twilight (see Glossary), whereas mice lacking both melanopsin and cone phototransduction retain robust photoentrainment (Altimus et al., 2010). In line with these data, spectral comparisons in retinally intact mice with red-shifted cone opsin sensitivity confirm that the ability of the circadian system to accurately measure low light levels (<10^11^ photons cm^−2^ s^−1^) can be entirely accounted for by rods (Lall et al., 2010).

Although a major role for rods in circadian photoentrainment under scotopic conditions (see Glossary) is not unexpected, the cost of this high sensitivity is that saturation in the rod phototransduction pathway limits its ability to provide information about higher levels of ambient illumination. A wide range of data now indicates that melanopsin provides the main source of information about ambient light levels across the range between civil twilight and full daylight (Fig. 2C; reviewed in Lucas et al., 2012). Thus, mice lacking rods and/or cones exhibit robust circadian responses under high light levels (Altimus et al., 2010; Freedman et al., 1999), whereas studies in cone-only and coneless mice indicate that cones do not contribute significantly to circadian responses under equivalent conditions (e.g. Freedman et al., 1999; Lall et al., 2010; Mrosovsky and Hattar, 2005; but see below).

In summary, mouse circadian responses based on measures of ambient light intensity almost entirely derive from a combination of rod and melanopsin photoreception. Currently, direct evidence confirming a similar role across other mammals is lacking. Nonetheless, melanopsin coding sequences are present in all vertebrates examined so far (Bellingham et al., 2006), suggesting a highly conserved role. Indeed, the spectral sensitivity of light-inhibited melatonin release (a surrogate marker of circadian photosensitivity) and its retention in individuals with advanced retinal degeneration (Czeisler et al., 1995) strongly suggest an important role for melanopsin in the human circadian system. However, the contribution of rods across mammalian species may be more variable.

Measureable circadian responses to very dim illumination have been reported in numerous night-active species (hamsters, rats, various murine rodents and bats), suggesting that rods contribute to photoentrainment in all nocturnal mammals (Daan and Pittendrigh, 1976; Joshi and Chandrashekaran, 1982; McGuire et al., 1973; Nelson and Takahashi, 1991; Takahashi et al., 1984). By contrast, circadian light sensitivity in humans seems to be at least two to three orders of magnitude lower than in mice, suggesting a less pronounced rod contribution (Gooley et al., 2010; Vartanian et al., 2015). Such data could indicate a generally reduced importance of rod-based circadian signals for diurnal mammals or may instead reflect the fact that rodent studies typically employ multiple days of dark adaptation that are impractical for use in humans (see below).

#### Cones, contrast and colour discrimination

Understanding the role of cones in circadian photoentrainment has proved challenging. As in most mammalian species, mice possess two classes of cone-opsin that are maximally sensitive to either short or longer wavelength light (S- and M-opsin, respectively; Jacobs and Williams, 2007). Although data from knockout mice suggest that cones play very little role in circadian responses under standard laboratory conditions (i.e. square light:dark cycles or long light pulses; Freedman et al., 1999; Lall et al., 2010; Mrosovsky and Hattar, 2005), the lighting employed by these studies is largely deficient in the UV light required to activate mouse S-opsin. As such, much of our information about the role of cones in circadian photoentrainment reflects just those that express M-opsin. Nonetheless, because most mouse cones express varying proportions of both S- and M-opsin (Applebury et al., 2000), these data have traditionally been interpreted as indicative of the role of cones in general.

There is also reason to expect that cones might not play a major role in circadian photoentrainment: to support high-acuity vision across the wide range of light levels encountered between twilight and bright sunlight, cone responses rapidly adapt under maintained illumination (Perlman and Normann, 1998). Consistent with this, studies using mice with altered cone spectral sensitivity indicate that although activation of M-opsin does influence circadian light responses, this effect is restricted to the first few minutes of exposure (Dkhissi-Benyahya et al., 2007; Lall et al., 2010). Thus, until recently it seemed that the unique contribution of cones to circadian photoentrainment was to allow the clock to respond to rapid changes in light intensity under photopic conditions (see Glossary; Lucas et al., 2012). However, new data indicate that this is only part of the story.

Although the properties of cones make them poorly suited for conveying information about brightness, the presence of spectrally distinct opsin subtypes allows most mammals to use cones to discriminate short- versus long-wavelength light (analogous to human blue–yellow colour vision; Jacobs, 2014). By providing information about changes in the colour of daylight around dawn and dusk (Fig. 2C,D), cones could thus provide information to the circadian system that is not available from rods or melanopsin. Because measuring colour relies on determining relative rather than absolute levels of photoreceptor activation, adaptation does not present the same barrier to measuring colour as it does for brightness. In addition, the resulting colour signal is less susceptible to environmental variations in illumination (e.g. due to clouds) that presumably constitute ‘noise’ for circadian measures of brightness. Accordingly, it is now clear that mice do use information about colour to adjust circadian timing (Walmsley et al., 2015). Indeed, under light cycles providing identical daily variation in rod and melanopsin activation, the inclusion of colour changes mimicking natural twilight are sufficient to produce very reliable effects on the timing of physiological rhythms.

Although this recent work shows that the mouse clock uses colour to obtain information about time of day, the exact effects of colour on circadian responses remain uncertain. Because later stages of twilight are associated with ‘bluer’ colours (Hulburt, 1953; Walmsley et al., 2015), the simplest scenario is that ‘blue’ light evokes weaker circadian responses than equally bright ‘yellow’ light. The data presented by Walmsley et al. (2015) support this view: mouse physiological rhythms (which require a daily phase delay to ensure a 24 h period) shift earlier when colour simulates a constant ‘late twilight’ as opposed to changing naturally around dawn and dusk. By contrast, other studies report that pulses of UV light (365 nm; appearing unnaturally ‘blue’ to mice) evoke phase delays of similar magnitude to longer wavelength stimuli (Provencio and Foster, 1995; van Oosterhout et al., 2012). Dissociating the contribution of colour under these conditions is challenging, however, because all mouse photoreceptors can respond to UV light (Fig. 2B). As such, more focused investigations of the specific effects of colour on mouse circadian photoentrainment are urgently required.

The contribution of colour to circadian responses in other mammals is currently unknown. A possible role in humans has been suggested based on acute changes in melatonin secretion evoked by polychromatic light (Figueiro et al., 2008, 2005, 2004). However, other studies have produced conflicting results (Gooley et al., 2010; Najjar et al., 2014; Papamichael et al., 2012; Revell et al., 2010). Moreover, given evidence that the spectral sensitivity of circadian responses deviates from that acutely controlling melatonin secretion (Gooley et al., 2010), the importance of colour for human circadian entrainment remains unresolved.

There is, however, extensive literature suggesting that non-mammalian species use colour to determine time of day. For example, the circadian period of the dinoflagellate *Gonyaulax* is directly sensitive to the amount of short- versus long-wavelength light (Roenneberg and Hastings, 1988). Similarly, colour discrimination mechanisms have been identified in the pineal or parietal organs of various fish and reptiles (Dodt and Scherer, 1968; Falcon and Meissl, 1981; Hamasaki, 1969; Jenison and Nolte, 1980; Uchida and Morita, 1994), providing a direct route through which colour could influence daily patterns of physiology and behaviour. There have also been suggestions that changes in colour alone may be sufficient to support circadian entrainment in certain fish and bird species (Pauers et al., 2012; Pohl, 1999). Unfortunately, without full details of the photoreceptive systems contributing to circadian entrainment in these organisms, it is hard to be certain that these reported effects of colour were not instead due to changes in apparent ‘brightness’ (e.g. see Fig. 2B). This distinction is especially important given the great diversity of extraretinal photoreceptors possessed by non-mammalian vertebrates (Peirson et al., 2009).

On balance, despite the complexity in unambiguously distinguishing the roles of colour in circadian entrainment, the data indicate a conserved use of colour as a time-of-day signal. It is tempting to speculate then that the utility of colour as a source of temporal information was one of the key selective pressures that drove the evolution of colour discrimination mechanisms. It is also, however, worth bearing in mind that a subset (∼10%) of mammals (including many marine and strongly nocturnal species) has lost the capacity for colour vision (Jacobs, 2013; Peichl, 2005). Clearly then, colour cannot provide equally valuable circadian information to all mammals. Presumably, in certain environments or for certain lifestyles, the increased temporal precision that measuring colour can provide does not convey any particular advantage.

## Neural underpinnings of circadian photoentrainment

**Fig. 3.**
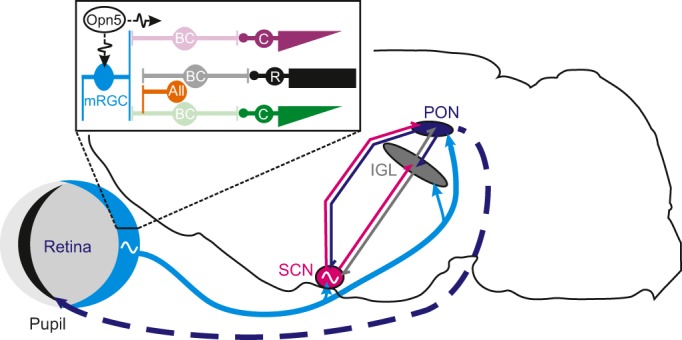
**Overview of the mammalian circadian visual system.** Neural control of mammalian circadian rhythms involves a widely distributed network with multiple control points. Three reciprocally interconnected retinorecipient nuclei in the brain contribute to the circadian response to light: the master circadian pacemaker in the hypothalamic suprachiasmatic nuclei (SCN), the thalamic intergeniculate leaflet (IGL) and the prectectal olivary nuclei (PON). Retinal input to all three regions (inset) arises primarily from melanopsin-expressing retinal ganglion cells (mRGCs), which are intrinsically photosensitive and also receive indirect input from rods and cones (R and C, respectively) via bipolar cells (BC) and AII amacrine cells (AII; see Glossary) (see Weng et al., 2013 for detailed discussion). Retinal output is also under direct control of a local circadian oscillator (represented by sine-wave symbol), dependent on neuropsin-expressing (Opn5) ganglion cells and indirectly influenced by the PON, which controls pupil aperture. Dashed line here represents a polysnaptic connection.

Although whole-animal assessments are essential for understanding the sensory signals used by the clock, the time required for these assessments and challenges in precisely controlling light exposure present substantial drawbacks. As such, studies of the neural circuitry that supports mammalian photoentrainment (Fig. 3) provide essential information for a detailed understanding of how photoreceptive signals are integrated within the clock and how findings in rodents will translate to other mammals.

### Retinal mechanisms

#### Properties of melanopsin-expressing retinal ganglion cells

Because the retina provides the sole source of photic input to the mammalian clock, an obvious starting point for understanding photoentrainment is to determine the visual response properties of the retinal neurons that provide this input. As discussed above, mRGCs provide the majority of the retinal input to the SCN and other relevant visual nuclei (Hattar et al., 2006) (see Fig. 3). Indeed, mRGCs were originally identified because they could be reliably found by retrograde tracing from the SCN (Berson et al., 2002), and they have now been identified in a wide range of species, including various nocturnal and diurnal rodents, cats and primates (Dacey et al., 2005; Gooley et al., 2001; Hannibal et al., 2002; Hattar et al., 2002; Jusuf et al., 2007; Karnas et al., 2013; Morin et al., 2003; Provencio et al., 2000; Semo et al., 2005).

Across all mammals examined so far, the morphology and projections of mRGCs are similar, indicating a conserved role in the circadian system. In mice and rats (where mRGCs have been investigated most extensively), there are five morphologically identified mRGC subtypes (Berson et al., 2010; Ecker et al., 2010; Estevez et al., 2012; Hu et al., 2013; Reifler et al., 2015; Schmidt and Kofuji, 2010). In these species, the subtype providing the majority of input to the SCN (M1 mRGCs) displays larger and more sensitive intrinsic light responses compared with the other subtypes, which primarily provide input to the visual thalamus and pretectum. It is unclear, however, whether there are other major functional differences between mRGC subtypes or whether possible functional specialisation of rodent M1 cells holds true also in primates (Liao et al., 2016).

In line with a role for rods in circadian responses to low light levels, mRGCs in primate and rodent retinas exhibit high-sensitivity rod-driven responses (Dacey et al., 2005; Weng et al., 2013; Wong et al., 2007). By contrast, presumably owing to the low density of melanopsin photopigment expressed by such cells, intrinsic mRGC photoreception requires much higher light levels (Do et al., 2009). Together, then, the distinct sensitivity ranges of rod and melanopsin phototransduction combine to allow mRGCs to linearly encode irradiance across a very wide range (>6 log units; Dacey et al., 2005; Weng et al., 2013; Wong et al., 2007). Moreover, mRGC responses display relatively little adaptation following extended illumination, allowing these cells to continuously signal irradiance for at least 10 h (Wong, 2012). This remarkable property may partly reflect the fact that melanopsin possesses an intrinsic bleach recovery mechanism (Box 1). In any case, the properties described above closely mirror those expected for a reliable source of ‘circadian brightness’ information.

Box 1. Bleach recovery and the bi- or tri-stability of melanopsin Conventional (ciliary) vertebrate opsins require 11-cis retinal for photoreception. Upon photon absorption, this is converted to all-trans retinal and then released. To regain photosensitivity (a process called ‘bleach recovery’), these opsins rely on an exogenous source of 11-cis retinal (provided by the retinal pigment epithelium). By contrast, melanopsin is more closely related to the rhabdomeric invertebrate opsins, which possess their own intrinsic bleach recovery mechanism – they are able to regenerate 11-cis retinal (Lucas, 2006). Thus, once activated, these ‘bistable’ invertebrate opsins can revert to their inactive state by absorbing a second (usually longer wavelength) photon (Koyanagi and Terakita, 2014). Such a mechanism was originally proposed to account for observations that long wavelength light seemed to sensitise melanopsin-driven responses (Mure et al., 2009, 2007; although see Mawad and Van Gelder, 2008). More recent evidence, however, suggests that melanopsin exists in one of three different stable states (i.e. it could be ‘tristable’), each exhibiting similar spectral sensitivity (λ_max_: 453–476 nm; Emanuel and Do, 2015; Matsuyama et al., 2012). Either bistability or tristability could, in principle, result in differences in the effective spectral sensitivity of melanopsin depending on prior light exposure. It is so far unclear, however, whether such effects significantly alter melanopsin sensitivity under real-world conditions; existing investigations suggest that the impact of bi- or tri-stability will be negligible under most commonly encountered sources of illumination (al Enezi et al., 2011; Brown et al., 2013).

The influence of cone pathways on the output of mRGCs is less certain. mRGCs do receive cone input, and one function of this seems to be to compensate for the sluggish kinetics of the intrinsic melanopsin-generated responses (∼20× slower than cone responses; Dacey et al., 2005; Do et al., 2009; Weng et al., 2013; Wong et al., 2007). In primates, however, at least some mRGCs receive opponent input from long (M/L-opsin) and short (S-opsin) wavelength-sensitive cone types, producing chromatic yellow-ON/blue-OFF signals (i.e. chromatic opponency, see Glossary; Dacey et al., 2005). By contrast, there is currently no evidence for chromatic responses in mouse mRGCs (Estevez et al., 2012; Hughes et al., 2013; Weng et al., 2013). It is worth noting, however, that existing attempts to define cone inputs to mouse mRGCs have relied on simply assessing responses to short- versus long-wavelength light (the interpretation of which is complicated by differences in apparent brightness). Also, notably, the chromatic mRGCs found in primates were identified based on projections to the thalamus and pretectum, not the SCN. It remains unclear, therefore, whether the influence of colour on mouse circadian timing (Walmsley et al., 2015) originates within a subtype of mRGCs and/or is shared with other mammalian species.

#### Retinal clocks

Other potential regulators of circadian photosensitivity are local retinal clocks. Indeed, although virtually all mammalian tissues contain intrinsic clocks, the retina is unique in being capable of photoentrainment in the absence of the SCN (Storch et al., 2007). Key properties and functions of the retinal clock have been discussed previously (McMahon et al., 2014), but most importantly here, there are daily variations in the function of all photoreceptive pathways: the speed and size of cone-based responses are enhanced during the day (Barnard et al., 2006; Cameron et al., 2008), the speed at which rods regain sensitivity (dark adapt) is enhanced during the night (Xue et al., 2015), and melanopsin expression and light response amplitude are similarly enhanced at night (Brown et al., 2011; González-Menéndez et al., 2009; Weng et al., 2009). Thus, the relative contributions of cone and melanopsin input to the circadian system could change throughout the day. More significantly, new data indicate that the photoreceptor(s) regulating these rhythms may differ from those used by the SCN (Box 2). Accordingly, the possible contribution of daily variations in retinal output is an important consideration for future studies seeking to understand how photoreceptor signals influence whole-animal circadian rhythmicity.

Box 2. Neuropsin photoreception and the retinal clock Recent data demonstrate that rhythms in retinal gene expression can be photoentrained even in mice lacking rods, cones and melanopsin (Buhr and Van Gelder, 2014). The photoreceptive origin of this entraining signal appears to be a UV-sensitive opsin protein, neuropsin, localised to a subset of mouse RGCs (Buhr et al., 2015) and expressed by many vertebrate species (Tarttelin et al., 2003; Yamashita et al., 2010). Remarkably, neuropsin is indispensible for photoentrainment of retinal gene rhythms (Buhr et al., 2015), suggesting that it either directly provides sensory information to the retinal clock or is an essential intermediary for other short-wavelength-sensitive pigments to do so. In either case, because light-dependent changes in RGC firing have not yet been reported in mice lacking rods, cones and melanopsin, it remains unclear how (or whether) light-dependent neuropsin activation could be communicated to other retinal neurons or the brain. Because behavioural photoentrainment is abolished in mice lacking rods, cones and melanopsin (Hattar et al., 2003), it certainly seems most unlikely that neuropsin can directly influence the central clock. Accordingly, mice lacking neuropsin display normal behavioural photoentrainment (Buhr et al., 2015). However, neuropsin might indirectly influence centrally driven circadian rhythmicity. For example, given their different sources of sensory control, the temporal relationship between daily rhythms in retinal and SCN output could vary under different lighting conditions.

### The central clock

Arguably the most promising way to define how the clock uses visual signals to estimate time of day is to determine how light modulates neuronal communication within the SCN itself. The basic timekeeping properties of the SCN are well understood: SCN neurons sustain intrinsic cycles of ‘clock gene’ expression that regulate downstream genes including ion channels and transporters, in turn driving pronounced rhythms in electrical output and excitability (for reviews, see Belle, 2015; Brown and Piggins, 2007; Colwell, 2011). Thus, in both diurnal and nocturnal mammals, SCN neurons are highly electrically active during the day and show lower activity during the night (Schwartz et al., 1983). Moreover, a subset of these SCN neurons are acutely light-responsive (Brown et al., 2011), and can therefore convey photic information to other cells in the SCN and beyond.

#### Properties of SCN neurons

**Fig. 4.**
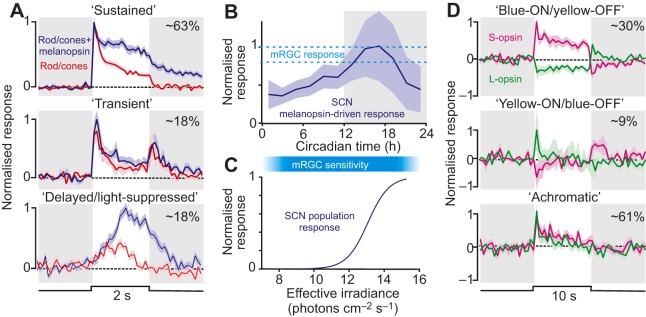
**Sensory properties of mouse SCN neurons.** (A) Characteristic response profiles of different classes of light-responsive SCN neurons in the mouse (normalised to maximal firing), revealed using stimuli that activate all classes of retinal photoreceptors (blue lines) versus those that strongly activate rods and cones but not melanopsin (red lines; means±s.e.m.; cells recorded as part of Brown et al., 2011). Note that although most cells receive both rod/cone and melanopsin input (‘sustained’), less commonly encountered cells show very little melanopsin response (‘transient’) or show sluggish increases or decreases in firing that are primarily driven by melanopsin (bottom panel). (B) The relative size of melanopsin-driven responses in ‘sustained’ SCN cells as a function of circadian time (mean±s.e.m.; derived from data in Brown et al., 2011) is a significantly larger change than observed in mRGCs themselves (range indicated by dashed lines; see Weng et al., 2009). (C) The sensitivity range of light-evoked SCN firing (for stimuli providing equal activation of all photoreceptor classes; Walmsley and Brown, 2015) is substantially narrower than that reported for mRGCs (represented by the blue bar; Dacey et al., 2005; Wong et al., 2007; Weng et al., 2013). (D) The diversity in cone-driven responses of SCN cells, determined using stimuli that selectively activate individual cone opsin classes (means±s.e.m.; based on data in Walmsley et al., 2015). A subset of SCN cells exhibit chromatic opponency, most commonly ‘blue-ON/yellow-OFF’ (top), whereas other cells show excitatory responses to activation of both cone opsin classes (bottom). For the plots shown in A, B and D, white backgrounds indicate periods of light and grey backgrounds indicate periods of darkness. For A and D, the percentage of cells that fall into each class is indicated in the upper right corner.

The basic properties of light-responsive SCN neurons have been assessed in various nocturnal (Brown et al., 2011; Meijer et al., 1986; Mure et al., 2007; Walmsley and Brown, 2015) and diurnal mammals (Groos and Mason, 1980; Jiao et al., 1999; Meijer et al., 1989). These experiments reveal a characteristic feature of the SCN light response: sustained increases or decreases in electrical discharge that are proportional to the intensity of illumination. Although these irradiance-coding properties broadly align with the properties of the mRGCs that provide this input, several features are worth noting (Fig. 4). Firstly, the sensory properties of SCN neurons seem surprisingly diverse relative to those of mRGCs (Fig. 4A). For example, the presence of light-suppressed cells is surprising, given that mRGCs are exclusively light activated and provide excitatory glutamatergic input to the SCN (Gompf et al., 2015). Secondly, there is a pronounced rhythm in the amplitude of light-evoked SCN activity that cannot simply be explained by daily variations in mRGC input (Fig. 4B; Brown et al., 2011; Meijer et al., 1996, 1998; van Oosterhout et al., 2012), presumably reflecting the intrinsic changes in SCN cell excitability indicated above. Finally, light-dependent modulation of SCN activity seems to require substantially (∼1000-fold) higher levels of illumination than that evoking measureable responses from mRGCs (Fig. 4C) (e.g. compare Brown et al., 2011; Weng et al., 2013).

Importantly, higher levels of light are also required to modulate SCN activity than to influence behavioural rhythms (Brown et al., 2011; Lall et al., 2010; Meijer et al., 1992). One obvious explanation here is that the procedures used to examine photic responses of SCN neurons (typically in anaesthetised animals that have not experienced lengthy periods of dark adaptation) render scotopic responses hard to detect. In fact, however, SCN light sensitivity in freely moving rodents is broadly similar to that of anaesthetised animals, even under near-constant darkness (Meijer et al., 1996, 1998; van Diepen et al., 2013; van Oosterhout et al., 2012). Although the impact of very long-term dark adaptation remains unexplored, at present it seems that rods actually evoke very little change in SCN neuronal activity. This is supported by investigations of the spectral sensitivity of the SCN light response (Aggelopoulos and Meissl, 2000; Brown et al., 2011). Although rod signals are detectable following prolonged dark adaptation, their impact on SCN firing is very small relative to the changes observed under photopic conditions. Indeed, the apparent mismatch between SCN and behavioural sensitivity to light may simply reflect the photon-counting properties of the SCN. Although the impact of rods on SCN firing is small, if sustained for many minutes, this could be enough to evoke a measureable effect on clock phase.

Consistent with this view, under conditions where light-evoked SCN activity can be easily measured, phase-shift magnitude is directly proportional to the amount of SCN activity evoked (Brown et al., 2011; Lall et al., 2010). Although it remains to be determined whether this observation reflects a causal link between spike firing and phase resetting, direct optogenetic manipulation of SCN firing rates appears to be sufficient to reset the clock (Jones et al., 2015). Moreover, many of the sensory properties observed for the mammalian circadian system are clearly reflected in the acute light-driven activity of SCN neurons: the sharp increases in firing displayed by SCN neurons at light onset primarily originate with cone photoreceptors, whereas sustained components of their responses are heavily dependent on melanopsin (Aggelopoulos and Meissl, 2000; Brown et al., 2011; Drouyer et al., 2007; Mure et al., 2007). Accordingly, stimuli that strongly activate mouse M/S-opsin co-expressing cones but not melanopsin evoke comparatively transient responses from SCN neurons (Fig. 4A; Brown et al., 2011), consistent with the observed effects of equivalent stimuli on whole-animal circadian timing (Lall et al., 2010).

By contrast, diversity in the sensory properties of individual SCN cells suggests that the relationship between SCN activity and phase resetting cannot be quite as simple as suggested above. Hence, a subset of SCN cells (15–20%) seems to entirely lack melanopsin-driven responses, and a similar proportion shows very little cone-driven response (Brown et al., 2011). The former group presumably receive input from non-melanopsin-expressing RGCs (Sollars et al., 2003), whereas the latter are assumed to reflect cells whose activity is indirectly driven by other light-sensitive neurons. Indeed, cells with similar properties are found in brain regions devoid of retinal input (Allen et al., 2016; Sakhi et al., 2014).

Most significantly, however, there is further diversity even among SCN cells that receive both cone and melanopsin input (Fig. 4D). Hence, although the majority of these cells is excited by selective activation of both cone opsin classes, ∼25% instead show chromatic opponency (Walmsley et al., 2015). Intriguingly, unlike the mRGCs identified in primates (Dacey et al., 2005), most of the chromatic cells in the mouse SCN exhibit blue-ON/yellow-OFF responses. The origins of these chromatic signals are currently unknown. Nonetheless, these data provide a clear mechanism by which changes in the colour of daylight can influence mouse circadian timing. The heterogeneity outlined above does, however, raise the question of how signals from the various SCN cell types are integrated to estimate time of day.

#### Local network processing

**Fig. 5.**
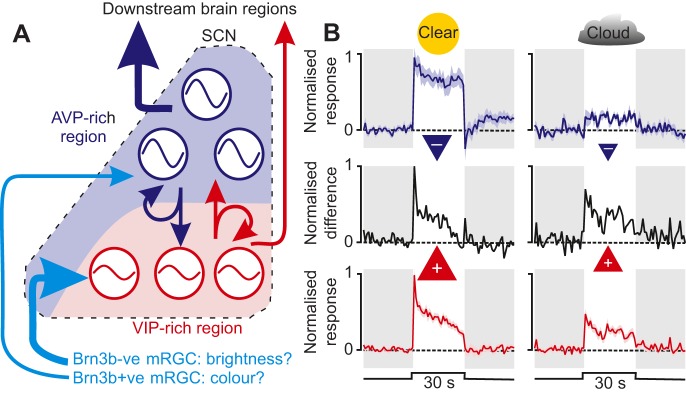
**SCN network organisation and possible implications of sensory processing.** (A) A proposed model of SCN organisation comprising reciprocally (and locally) coupled vasoactive intestinal polypeptide (VIP)- and arginine-vasopressin (AVP)-rich regions (red and blue, respectively). The VIP-cell region receives dense retinal input (thick light blue arrow) and contains weak oscillators (represented by shallow waves) that are easily reset by external stimuli. The AVP-cell region receives less retinal innervation (thin light blue arrow), but contains more robust oscillators (deeper waves) and provides more extensive innervation of downstream brain regions (compare thick dark blue arrow versus thin red arrow). Retinal input to the two regions derives from genetically distinct mRGC subtypes, which are either Brn3b positive or negative (Brn3b+ve or −ve, respectively), proposed here to reflect cells providing chromatic versus achromatic input, respectively. (B) The normalised (relative to maximal firing) activity of blue-ON cells (top) and achromatic cells (bottom) to light steps replicating the colour and brightness of natural daylight (solar elevation=+3 deg) on clear (left panels) or cloudy days (right panels). Red and blue triangles represent the approximate strength of proposed excitatory (+) and inhibitory (−) contributions to phase resetting. Middle panels in B represent the difference between achromatic and blue-ON cell activity (weighted according to the twofold greater density of achromatic cells). Note that, because blue-ON cell activity is much more strongly suppressed under cloudy days than that of achromatic cells, the difference in activity of the two populations for a fixed solar elevation is broadly similar regardless of weather conditions. Periods of light are represented by white backgrounds and periods of darkness are represented by grey backgrounds. Data in B are derived from Walmsley et al. (2015).

The basic anatomical organisation of the SCN is highly conserved across mammals, with a common set of inputs and several neurochemically identifiable cell types (Cassone et al., 1988; Moore, 1993; Morin and Allen, 2006), most notably, those producing vasoactive intestinal polypeptide (VIP) or arginine-vasopressin (AVP). The generally accepted model of how these elements interact (Fig. 5A) suggests that the SCN comprises two reciprocally coupled populations: a weakly rhythmic retinorecipient population that is highly responsive to external signals (primarily VIP cells) and a strongly rhythmic population (primarily AVP cells) that provides the major source of clock output and is more resistant to external perturbation (Evans and Gorman, 2016; Meijer et al., 2010). Although a full account of the evidence for and against this model is beyond the scope of this Review, there are a few aspects of SCN organisation worth discussing that are relevant to how sensory signals may be processed within the SCN.

Firstly, the SCN receives strongly bilateral retinal innervation (Cassone et al., 1988; Moore, 1993; Morin et al., 2003, 2006; Morin and Studholme, 2014) relative to other retinorecipient nuclei (especially in laterally eyed mammals, where ipsilateral retinal input is restricted to the small zone of binocular overlap). Indeed, at least in the hamster, each SCN receives input from across the full extent of both retinas (Morin et al., 2003). This is presumed to allow the SCN to measure the average amount of light across the full visual field. Recent work in mice, however, indicates that integration does not occur at the level of individual cells (Walmsley and Brown, 2015). Instead, SCN cells measure brightness from, at most, one visual hemisphere and possibly just a small portion of the retina, as reported in an early investigation of the cat SCN (Groos and Mason, 1980). Because of this arrangement, light detected by one eye activates half the number of SCN neurons as the same amount of light split across both eyes (Groos and Mason, 1980; Muscat and Morin, 2005; Walmsley and Brown, 2015). As such, it seems that activity of the SCN network also does not, strictly speaking, report the average amount of environmental light, and is instead disproportionately sensitive to diffuse illumination. A key question for the future will be to determine whether these observations translate to an equivalent bias in circadian phase-resetting responses at the whole-animal level.

Secondly, in mice, both the VIP and AVP cell populations receive retinal input (Lokshin et al., 2015) but from different sources: input to the AVP-expressing region derives from mRGCs that express the transcription factor Brn3b, whereas the more common Brn3b-negative mRGCs innervate the VIP-rich region (Chen et al., 2011). Although it is not known whether these genetically distinct mRGC subtypes convey different signals, it is tempting to speculate that it is the minority Brn3b-positive population that provides chromatic information. Indeed, blue-ON cells appear to be preferentially located in the AVP-cell rich portions of the SCN that receive this input (Walmsley et al., 2015). By contrast, behavioural studies have so far failed to identify a role for Brn3b-positive mRGCs in photoentrainment (Chen et al., 2011), although the conditions tested were not well suited to identify the contributions of cones or colour.

In any case, the implication of the hypothesis above is that neuroanatomically defined SCN cell populations might convey quite different sensory signals (colour versus brightness). Because AVP and VIP cells may differentially innervate SCN target nuclei (Abrahamson and Moore, 2001; Canteras et al., 2011; but also see Watts and Swanson, 1987; Morin, 2013), this might allow the clock to independently tune the daily signals conveyed to particular target regions. Alternatively, this might provide a clue as to how colour and brightness signals are integrated within the SCN clockwork. Hence, AVP-receptor knockout mice exhibit enhanced clock resetting, suggesting that AVP cell activity inhibits phase-shifting responses (Yamaguchi et al., 2013). If such cells convey blue-ON signals, this could explain why conditions that increase blue-ON cell activity seem to provide a weaker entrainment signal (Walmsley et al., 2015). Such a mechanism could then compensate for the ambiguous information about sun position available from measuring brightness, by disinhibiting circadian responses to unexpectedly low levels of daytime illumination on overcast days (where blue-ON cell activity is strongly suppressed; Fig. 5B). Direct tests of these predictions constitute important challenges for future research.

### Modulatory inputs

The two other sources of visual signals that could modulate sensory processing within the SCN are the thalamic intergeniculate leaflet (IGL, homologue to the primate pregeniculate nucleus) and the prectectal olivary nuclei (PON). These regions are reciprocally connected with one another and with the SCN (Mikkelsen and Vrang, 1994; Moga and Moore, 1997; Morin and Blanchard, 1998). Moreover, both of these regions receive substantial mRGC input, albeit with more significant contributions from non-M1 cells and potentially also non-melanopsin-expressing RGCs (Baver et al., 2008; Chen et al., 2011; Ecker et al., 2010).

#### Intergeniculate leaflet

**Fig. 6.**
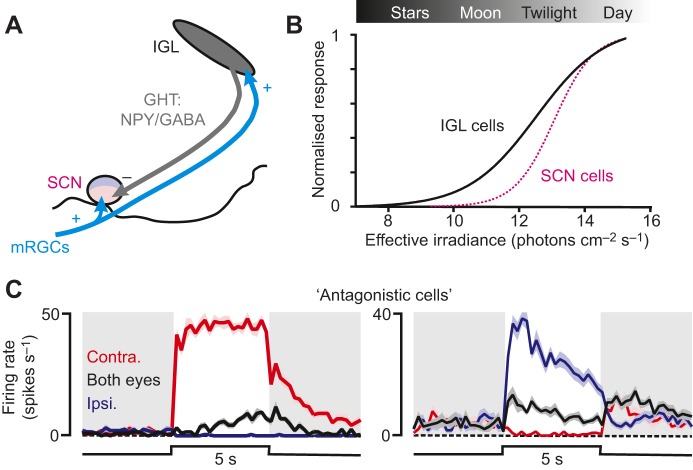
**Geniculohypothalamic inputs to the central clock.** (A) Schematic of the geniculohypothalamic pathway. The IGL receives dense excitatory input (+) from mRGCs, and GABAergic neurons in this region [including a population that co-expresses neuropeptide Y (NPY)] provide inhibitory input (−) to VIP-rich portions of the SCN. (B) The normalised (according to maximal firing) sensitivity of cells in the mouse IGL region to light steps providing equal activation of all photoreceptor classes. The data reveal substantially larger responses to low light levels than those shown by SCN cells tested under equivalent conditions. (C) Responses of ‘antagonistic’ cells in the IGL region (similar to those reported for some SCN-projecting cells) that selectively respond to illumination of just one eye, with little response to binocular illumination. Periods of darkness are indicated by grey backgrounds and periods of light are indicated by white backgrounds. IGL data in B and C are derived from data presented in Howarth et al. (2014) and SCN sensitivity from Walmsley and Brown (2015).

IGL cells send inhibitory GABAergic projections to the SCN [Fig. 6A; the geniculohypothalamic tract (GHT)]; these projections include a population of cells that co-express neuropeptide Y (NPY; Harrington, 1997; Morin and Allen, 2006). In nocturnal rodents, electrical activation of the GHT (or intra-SCN microinjection of NPY) exerts phase-resetting effects that are opposite to those of light (Huhman and Albers, 1994; Rusak et al., 1989). Importantly, however, although this pathway is known to mediate circadian responses to arousal-inducing stimuli (Janik and Mrosovsky, 1994; Johnson et al., 1988; Marchant et al., 1997; Wickland and Turek, 1994), its role in photoentrainment remains elusive.

Pharmacological studies suggest that release of NPY from the GHT may actively oppose circadian light responses (Lall and Biello, 2002, 2003; Yannielli et al., 2004; Yannielli and Harrington, 2000). By contrast, although there is disagreement as to the precise effects of IGL lesions (Harrington and Rusak, 1986; Maywood et al., 1997; Muscat and Morin, 2006; Pickard et al., 1987), on balance these studies suggest that hamster circadian responses to light are reduced in the absence of a functional GHT. Considering data from these and other lesion studies, perhaps the clearest hypothesis that can be advanced is that the GHT is required for normal temporal integration of photic information (Edelstein and Amir, 1999; Evans et al., 2012; Johnson et al., 1989; Morin and Pace, 2002; Pickard, 1994).

Given the inhibitory nature of this projection, it is surprising that the weight of evidence indicates positive contributions of the GHT to circadian photoentrainment. Indeed, as one would expect based on the extensive mRGC input, most IGL cells exhibit irradiance-dependent increases in firing rate (Brown et al., 2010; Harrington, 1997). Of course, it is possible that IGL lesion studies do not fully reveal the physiological roles of this projection (e.g. because of non-specific damage of the shared light input pathway; Morin et al., 2003; Pickard, 1985) and/or that the lighting conditions tested to date were not suited to reveal inhibitory actions of the GHT. For example, thalamic neurons targeted by mRGCs appear to respond more robustly under scotopic conditions than do SCN cells (Fig. 6B; Brown et al., 2011; Howarth et al., 2014; Muscat and Morin, 2006; Walmsley and Brown, 2015). Thus, one possible function of the GHT could be to suppress circadian responses to very low light levels, as suggested by one previous lesion study (Evans et al., 2012).

Alternatively, because not all IGL cells contribute to the GHT (Moore and Card, 1994; Morin and Blanchard, 2001), the properties of GHT cells might differ from those of most IGL neurons. A few studies have directly identified GHT cells by stimulating their SCN terminals (Blasiak and Lewandowski, 2013; Harrington and Rusak, 1989; Zhang and Rusak, 1989) or indirectly inferred their properties by identifying NPY cells (Thankachan and Rusak, 2005). Although none of these studies involved detailed sensory characterisation, collectively they suggest that the GHT derives from a mixed population of cells including those that are activated, suppressed or apparently unresponsive to light. Perhaps most interestingly, a subset of GHT cells combines binocular signals in an antagonistic manner (Harrington and Rusak, 1989; Zhang and Rusak, 1989), an unusual property that is particular to a group of cells in the IGL and neighbouring thalamus (Howarth et al., 2014) and that renders them preferentially sensitive to inhomogeneous illumination of the two eyes (Fig. 6C).

We can currently only speculate about the functional role that such ‘antagonistic’ cells might play in circadian photoentrainment. It is worth considering, however, the situations in which antagonistic cells are likely to be most active. One such condition is when changes in head or eye position mean that a much larger proportion of one retina is directed at the sky relative to the opposing eye. Antagonistic binocular signals could, conceivably, help the clock adjust its assessment of brightness under such conditions. This would imply a surprising degree of sophistication of sensory processing within the circadian visual network. Remarkably, however, there is some evidence that supports a role of this kind: IGL cells integrating vestibular and associated visual signals have been identified previously (for a review, see Harrington, 1997), and neuroanatomical tracing suggests the presence of a polysynaptic projection from vestibular nuclei to the SCN that runs via the GHT (Horowitz et al., 2004).

#### Pretectal olivary nuclei

The PON is best known as the primary central relay controlling pupil constriction (Gamlin, 2006). Despite reciprocal connections with both the IGL and the SCN (Moga and Moore, 1997; Morin and Blanchard, 1998), a direct contribution of the PON to circadian photoentrainment has not yet been investigated. Nonetheless, because the pupil regulates the amount of light entering the eye and can thus indirectly alter circadian assessments of brightness (∼100-fold for a fully constricted versus dilated pupil in the mouse), some discussion of the PON is relevant here.

As described for SCN neurons, the sensory properties of PON cells and pupillary constriction primarily originate from a combination of cone and melanopsin signals (Allen et al., 2011; Gamlin, 2006; Gamlin et al., 2007; Lucas et al., 2001, 2003; McDougal and Gamlin, 2010; Spitschan et al., 2014), with cones accounting for early components of the responses to changes in illumination (first few seconds) and melanopsin becoming increasingly important for later components. However, in line with the properties of primate PON-projecting mRGCs (Dacey et al., 2005), recent investigations reveal a yellow-ON/blue-OFF influence on the human pupil (Spitschan et al., 2014). Control of pupil size thus provides at least one route by which colour could influence the primate circadian system. By contrast, although influences of colour on rodent pupil responses are unknown, it seems unlikely that these will directly recapitulate results from humans. Indeed, mouse PON neurons exhibit sustained increases in firing that involve a substantial contribution from S-opsin biased cones (Allen et al., 2011). Accordingly, if the mouse pupil is under chromatic control, it is most likely of the blue-ON variety.

In either case, it is important to consider the extent to which sensory properties of the pupillary system could influence those of circadian photoentrainment. Because light levels evoking pupillary and circadian responses in humans largely overlap (Gamlin et al., 2007; Gooley et al., 2010), such an influence is potentially significant. By contrast, a study in hamsters concluded that the substantially higher sensitivity of circadian responses ensures that an influence of pupil constriction is not functionally relevant in this species (Hut et al., 2008). However, the conditions used for that study are likely to favour high-sensitivity circadian responses. Under alternative conditions (e.g. around dusk or for very brief light exposure), one might expect a greater overlap. Moreover, there certainly seems to be more extensive overlap between circadian and pupillary sensitivity in other rodents such as mice (Lall et al., 2010). Finally, it is worth noting that there is evidence for circadian modulation of pupil responsiveness (with greater constriction during the projected day) in both mice and humans (Münch et al., 2012; Owens et al., 2012). Potential influences of pupil constriction should thus be carefully considered in any whole-animal investigation of the sensory control of circadian rhythmicity.

## Conclusions and implications

As highlighted above, the organisation and properties of the mammalian circadian system are remarkably similar regardless of what ecological niche an organism occupies. Nonetheless, sensory regulation of circadian photoentrainment is complex, with multiple processing steps that are not all well understood but could produce differences in the properties of the circadian light response based on time of day and light exposure history, and/or between species. There are, however, two basic features that seem to be nearly universally true: (1) a temporal gating mechanism ensures that circadian sensitivity to light is maximised around dawn and dusk and (2) the principal source of light input to the clock comes from mRGCs, which combine signals from rods and cones with their intrinsic melanopsin-derived light response.

A combination of rod and melanopsin signals is expected to provide an important source of information about circadian brightness for all mammals. Although the relative contributions of these photoreceptors is not necessarily a fixed property, their similar spectral sensitivity across mammalian species (al Enezi et al., 2011; Bailes and Lucas, 2013; Berson et al., 2002; Dacey et al., 2005; Hisatomi and Tokunaga, 2002) makes predicting the apparent magnitude of this brightness signal relatively straightforward. A more complex question is how cones contribute to circadian photoentrainment. In mice, it seems that cones can play a dual role: allowing the clock to track sudden changes in light intensity across the twilight–daytime transition, while also providing information about slower changes in the colour of daylight. The extent to which this represents the role of cones in other mammals is still unclear. Insofar as the relationship between colour and time of day is broadly similar for all mammals, it seems likely that any organism capable of extracting such information would make use of it. Nonetheless, not all mammals are capable of colour vision and, among those that are, the spectral sensitivity of short- and longer-wavelength-sensitive cones can differ substantially (intrinsically and/or because of differences in lens transmission; Hut et al., 2012; Peichl, 2005). Thus, the magnitude of the apparent colour change across twilight will vary between species. The higher proportion of cones in the retina of diurnal mammals, relative to nocturnal species, may also increase the importance of cones as a source of circadian colour and/or ‘brightness’ information for these species (e.g. Gooley et al., 2010). As such, detailed comparative studies of how cones and colour influence mammalian circadian photoentrainment are urgently required.

In the interim, it is still possible to draw some conclusions as to how man-made alterations in the environment might impact upon our own clocks or those of animals living in urban environments. Typical levels of nocturnal illumination in cities and towns (∼10 lux) are, for the human photopic visual system, at least 10 times what one would naturally experience on a full moonlit night (Cinzano et al., 2001). Depending on the source of such illumination, this equates to an effective rod or melanopsin signal of >10^12^ photons cm^−2^ s^−1^ (for fluorescent or LED lighting; Lucas et al., 2014). Based on the high-sensitivity circadian responses typically reported for nocturnal animals, illumination of this magnitude is sufficient to significantly disrupt appropriate biological timing. Indeed, there is already direct experimental evidence for such effects in mice as well as in diurnal rodents and birds (Bedrosian et al., 2011; Da Silva et al., 2015; Fonken et al., 2013, 2012, 2010). Our 24 h society and increasing reliance on artificial sources of illumination (∼2 orders of magnitude dimmer than natural daylight) also means that many of us may no longer be receiving sufficiently robust daily circadian timing signals (Wyse et al., 2014). Again, mounting evidence indicates that this is associated with a negative impact on health (e.g. Archer and Oster, 2015; Pan et al., 2011; West and Bechtold, 2015). In the future, a better understanding of the sensory control of the circadian clock in humans and animals (especially the impact of colour) should allow us to design more healthy environments that support optimal circadian alignment.
